# Supplementary material for: Activation of the Unfolded Protein Response (UPR) Is Associated with Cholangiocellular Injury, Fibrosis and Carcinogenesis in an Experimental Model of Fibropolycystic Liver Disease
Source: Cancers (Basel). 2021 Dec 24;14(1):78. doi: 10.3390/cancers14010078 (PMC8750579; doi:10.3390/cancers14010078)
Supplement: Supplementary file 1 [file cancers-14-00078-s001.zip › cancers-1515734-supplementary.pdf]

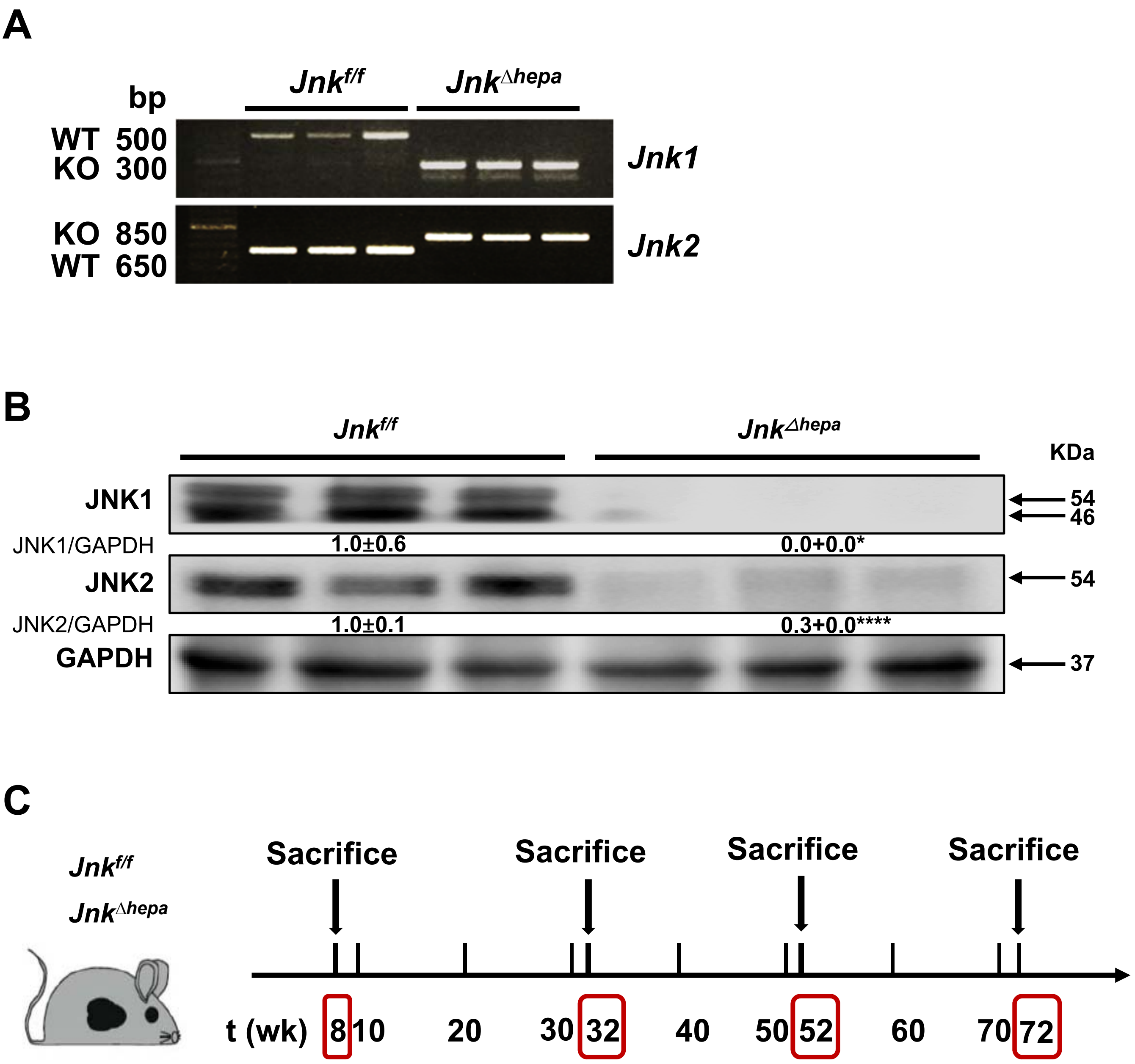

Figure S1. (**A**) Genotyping for Jnk1 and Jnk2. Ethidium Bromide gel showed the respective PCR results from mice tails DNA derived from JnkΔhepa mice. (**B**) Immunoblot analysis to demonstrate the deletion of JNK1 and JNK2. GAPDH was used as a loading control. (**C**) Experimental setting: Jnkf/f and JnkΔhepa mice were sacrificed at different time points of the progression of liver disease, 8, 32, 52 and 72 weeks.

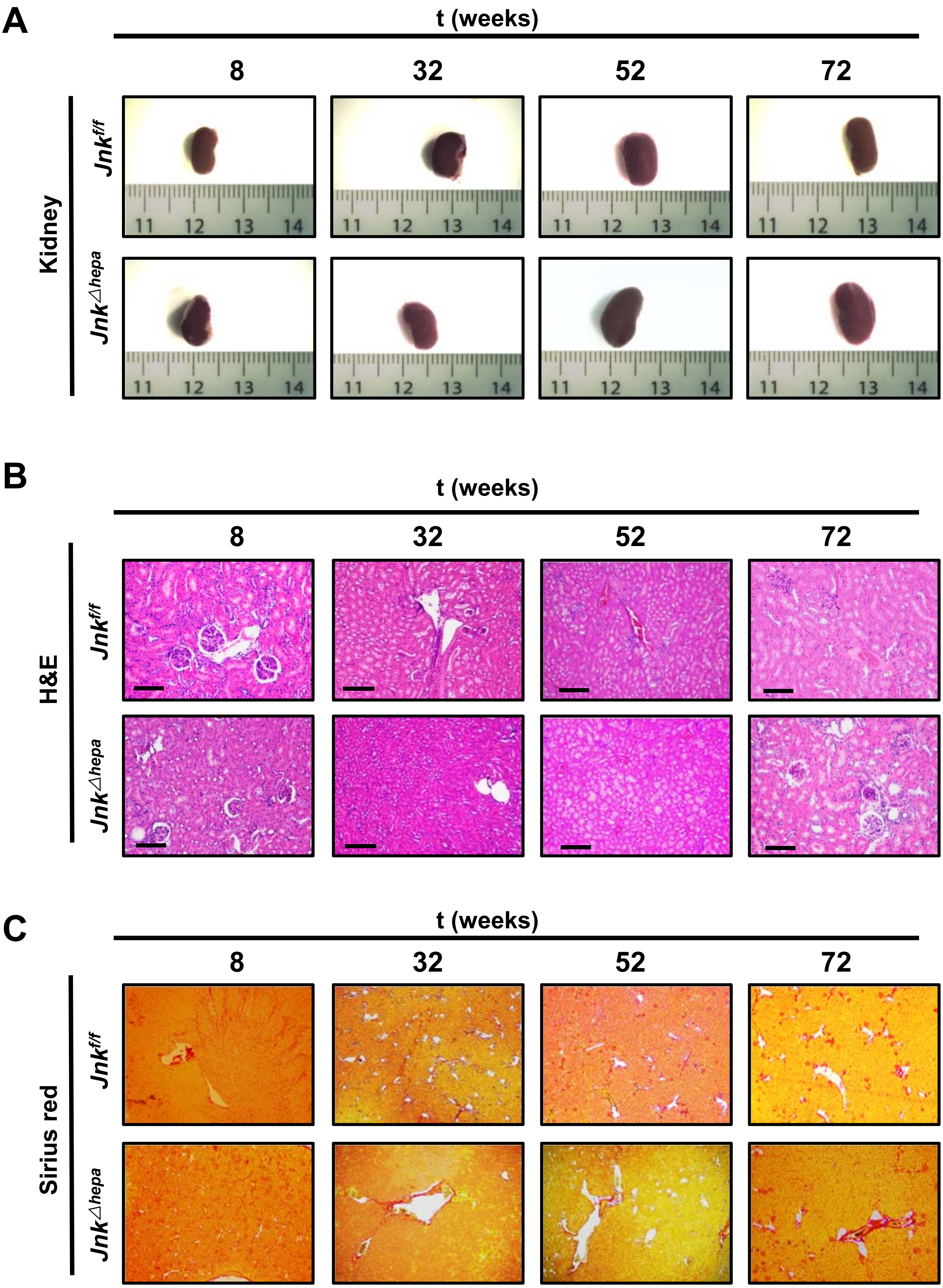

Figure S2. (**A**) Macroscopic view of the kidney of ageing Jnkf/f and JnkΔhepa mice. (**B**) H&E staining was performed in the kidneys of 8 to 72-week-old Jnkf/f and JnkΔhepa mice. Scale bars, 200µm. (**C**) Sirius Red (SR) staining was performed in the kidneys of 8 to 72-week-old Jnkf/f and JnkΔhepa mice. Scale bars, 100µm.

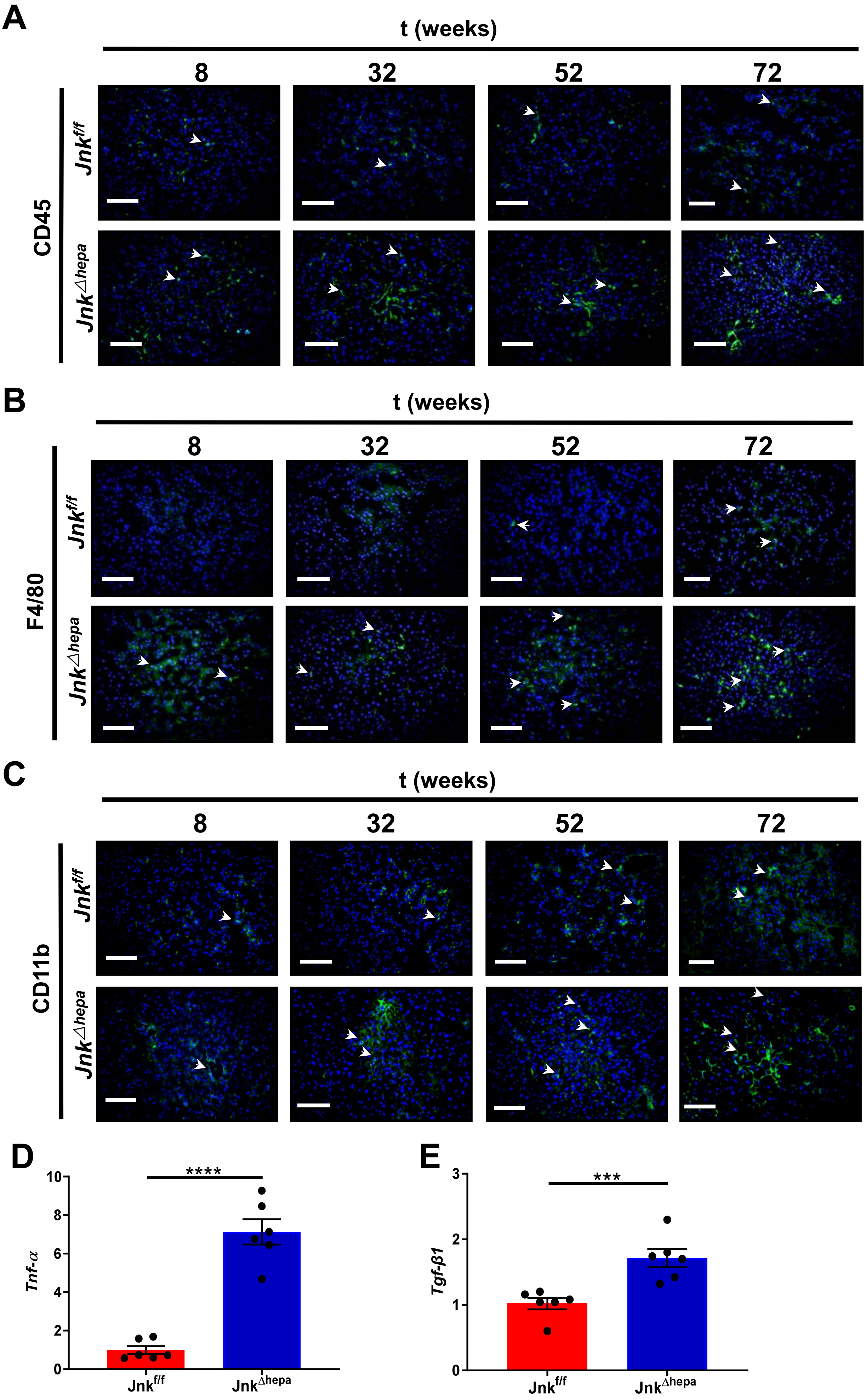

Figure S3. (**A**) Representative IF microphotographs of 8 to 72 week-old Jnkf/f and JnkΔhepa mice for CD45 (**B**), F4/80 (**C**), CD11b (**D**). Scale bar, 200 µm. The mRNA expression of Tnf (**E**) and Tgfβ1 (**F**) was calculated and graphed in 32 week-old Jnkf/f and JnkΔhepa mice (***P < 0.001, ****P <0.0001).

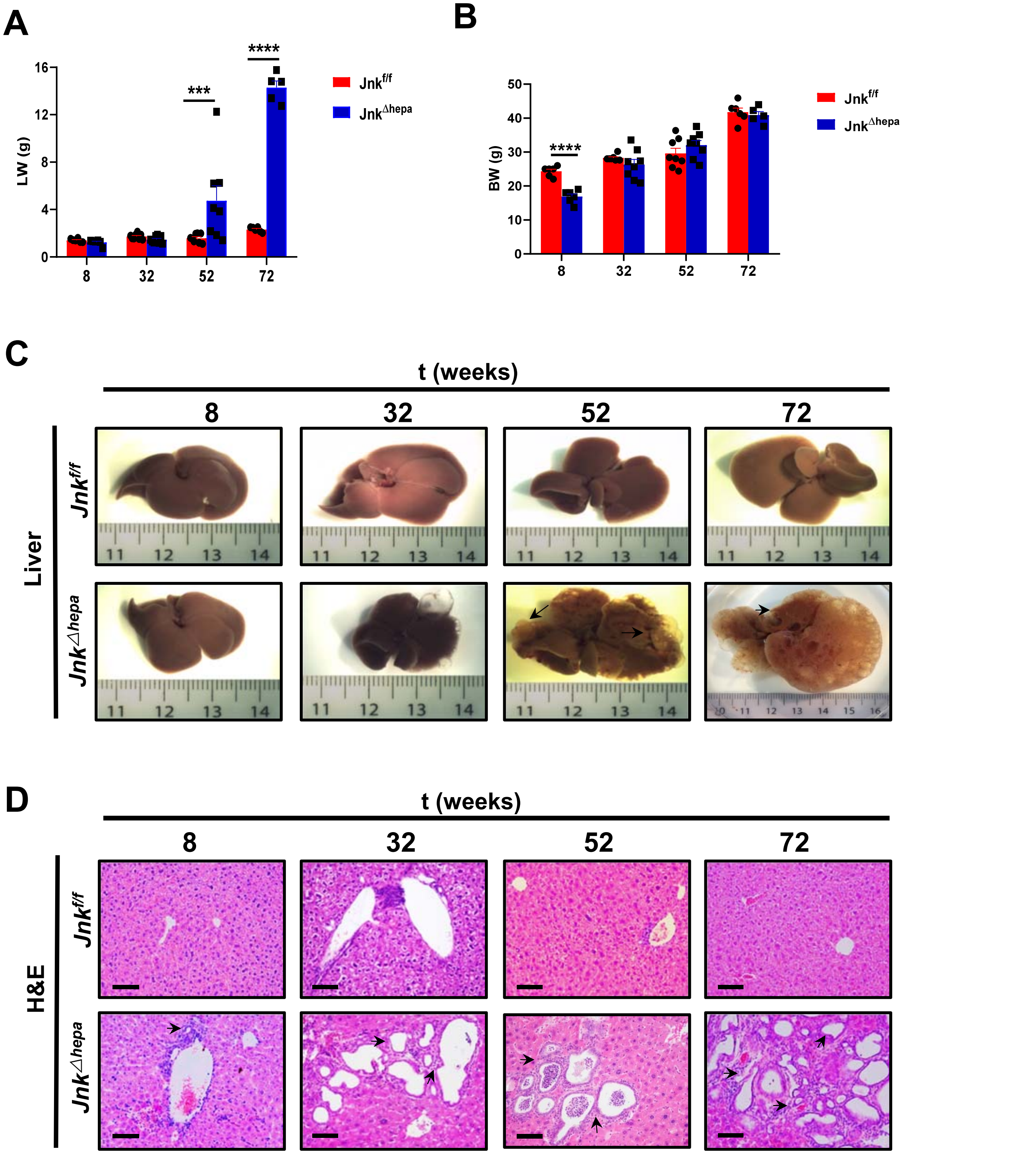

Figure S4. Liver weight, LW (**A**) and body weight, BW (**B**) were measured in ageing Jnkf/f and JnkΔhepa mice (mean ± SEM; n=6-8 per time point; **P < 0.01, ***P < 0.001, ****P <0.0001). (**C**) Macroscopic view of livers from ageing Jnkf/f and JnkΔhepa mice at 8, 32, 52 and 72 weeks of age. (**D**) H&E staining was performed in paraffin sections of the same livers.

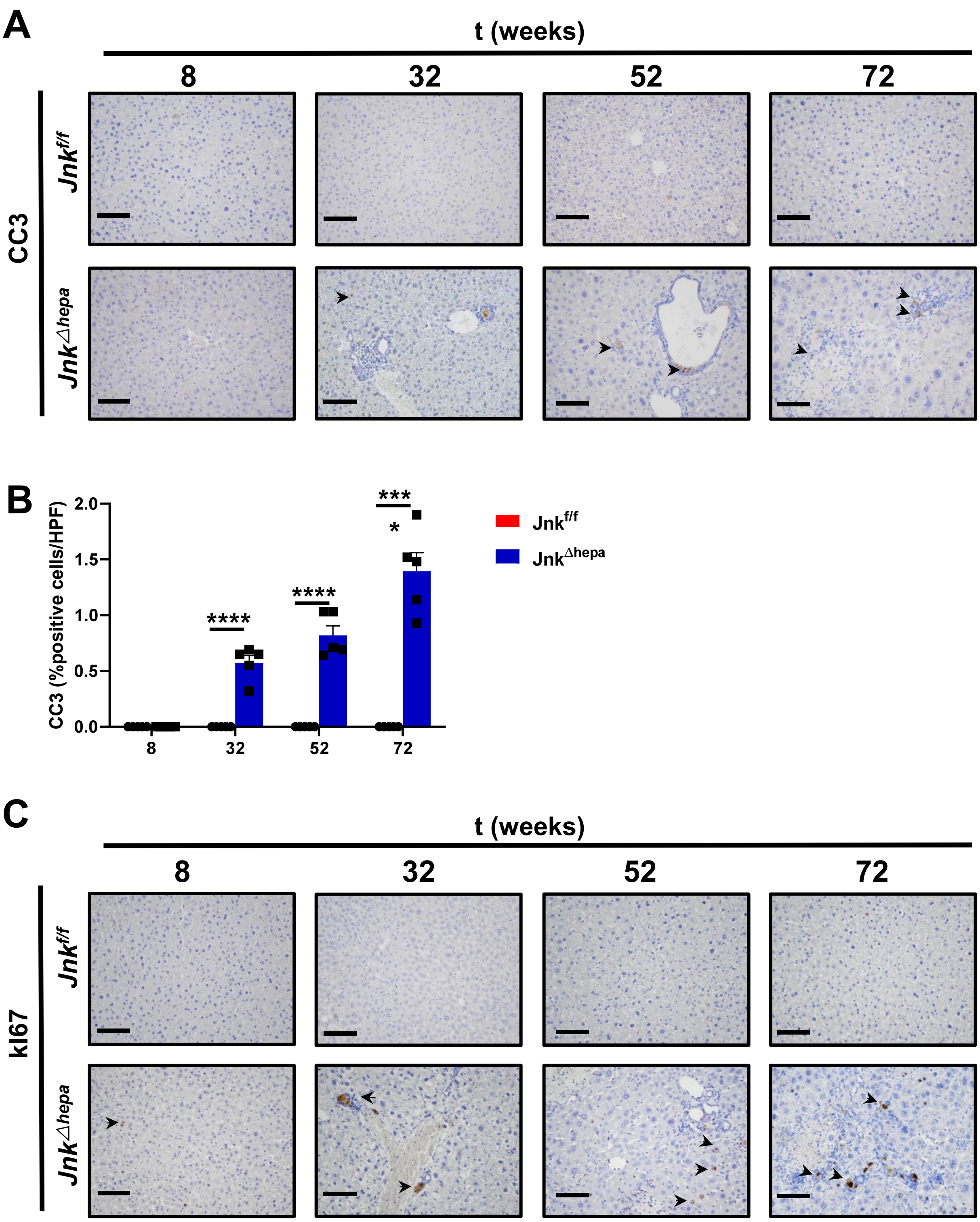

Figure S5. (**A**) Inmunohistochemistry staining for cleaved caspase 3 (CC3) was performed in liver sections of ageing Jnkf/f and JnkΔhepa mice, from 8 to 72 weeks of age. (**B**) Quantification of positive cells was measured and graphed (n=**P < 0.01, ***P < 0.001, ****P <0.0001). (**C**) Inmunohistochemistry staining for Ki67 was performed in liver sections of ageing Jnkf/f and JnkΔhepa mice, from 8 to 72 weeks of age.

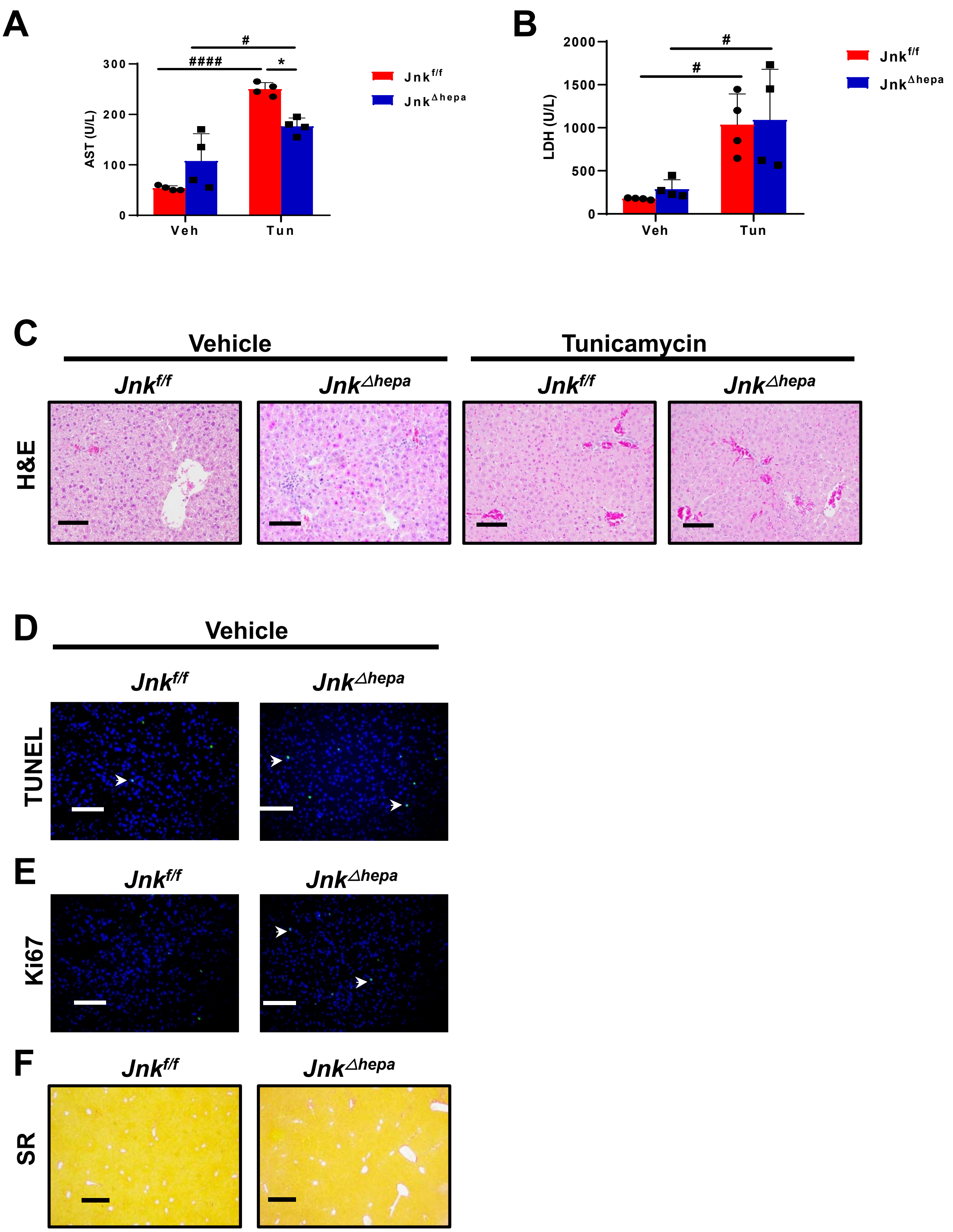

Figure S6. (**A**) Sirius Red (SR) was performed for 8 week-old vehicle-treated Jnkf/f and JnkΔhepa mice and microphotographs are shown. (**B**) TUNEL was assessed for the same mice and a microphotograph is shown. (**C**) Ki67 was performed in the same samples and a microphotograph is shown.

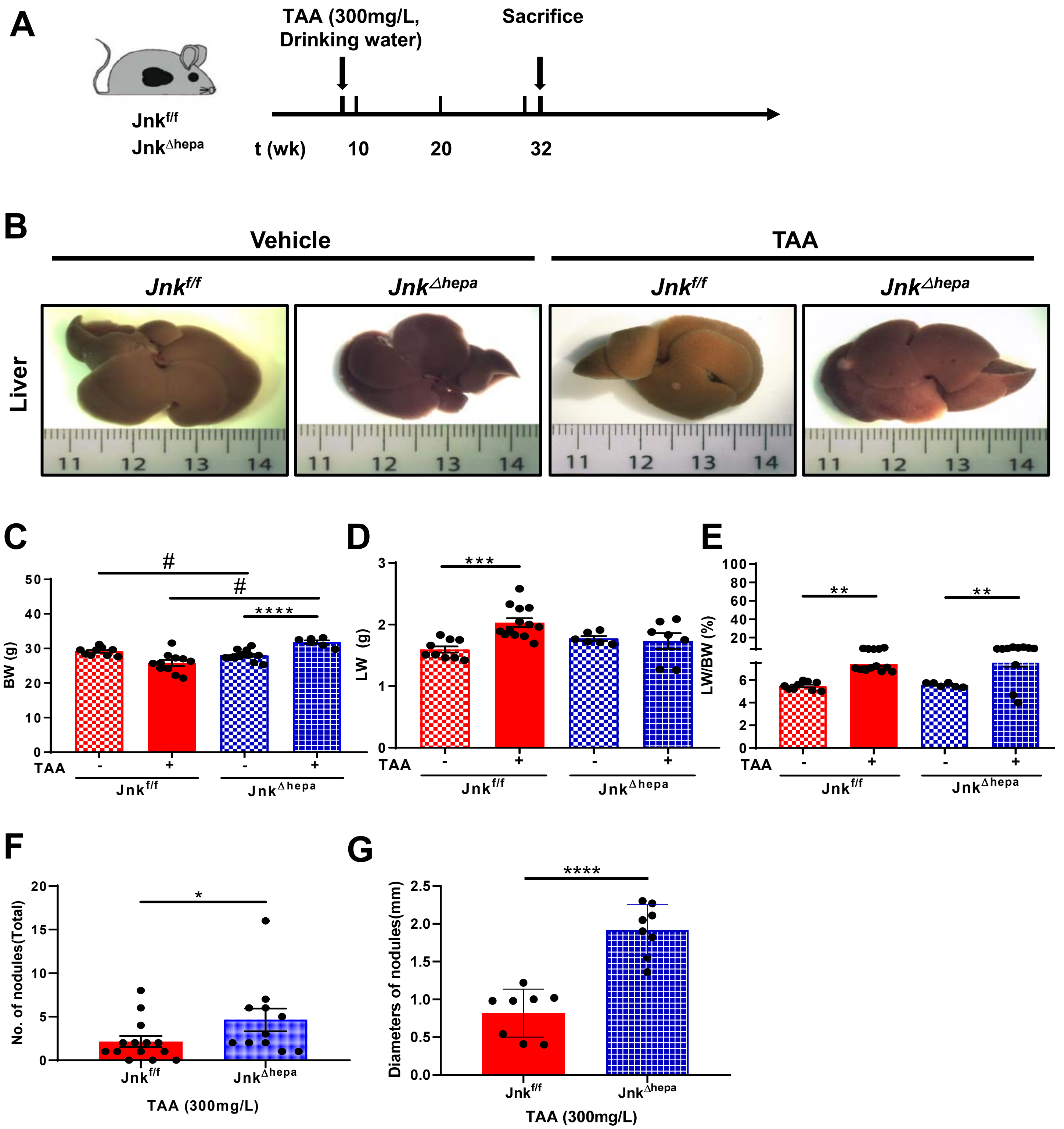

Figure S7. (**A**) Eight week-old Jnkf/f and JnkΔhepa male mice were used for administration of TAA in drinking water (300mg/L). Mice were sacrificed 24 weeks later (n=6-13). (**B**) Macroscopic pictures of the liver after sacrifice are shown. BW (**C**), LW (**D**) and LW/BW (**E**) ratio was calculated. (**F**) Total liver nodules were counted and represented after treatment. (**G**) Diameter of liver nodules was measured and graphed. Data are shown as mean ± SEM and graphed (*p<0.05; **p<0.01, ***p<0.001; intragroup and #p<0.05 intergroup comparison).

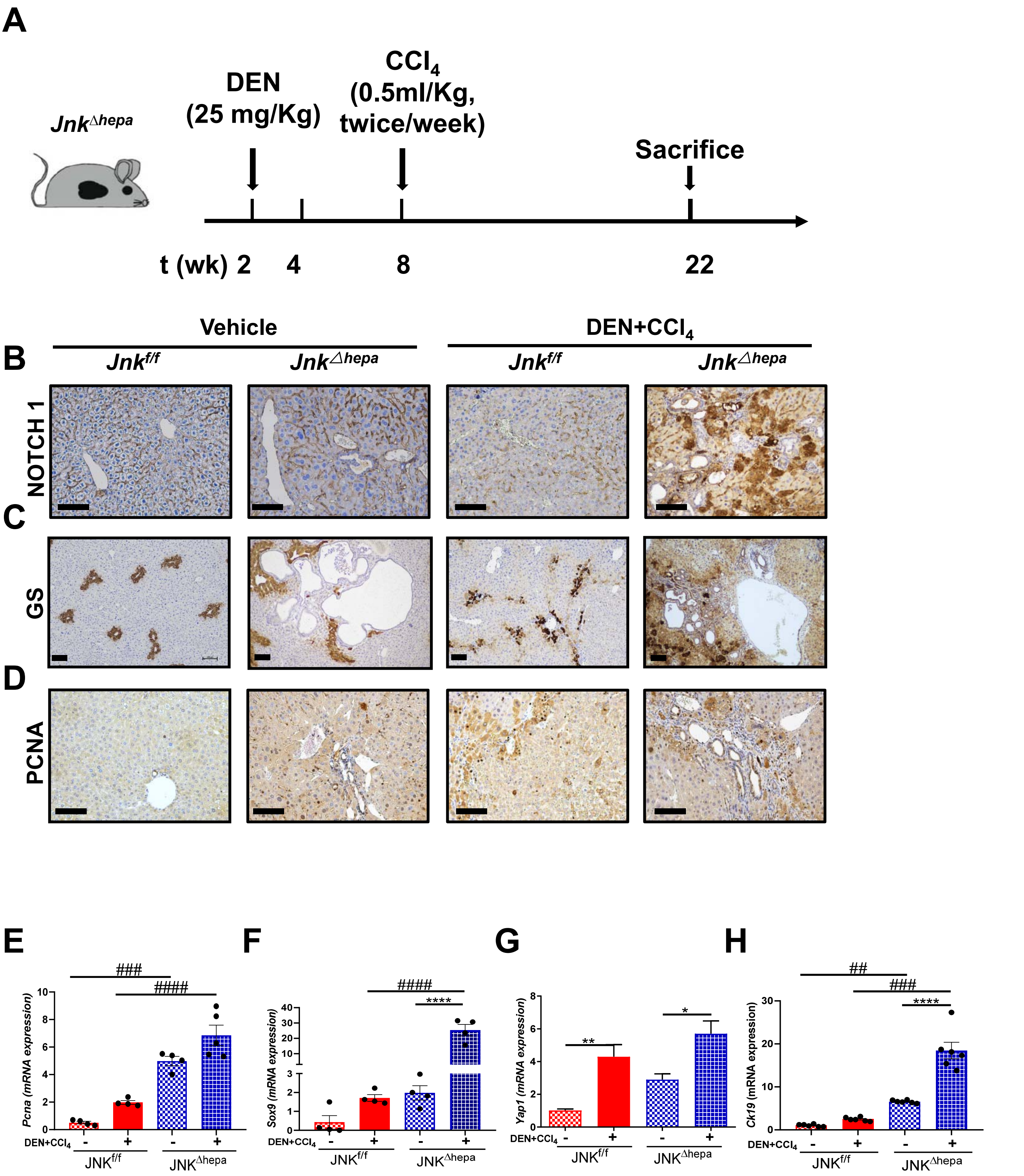

Figure S8. (**A**) Seven week-old Jnkf/f and JnkΔhepa male mice were used for administration of DEN/ CCl4. Mice were sacrificed 8 weeks after DEN injection (n=7). Notch1 (**B**), Glutamine synthase (GS) (**C**) and PCNA (**D**) immunohistochemistry staining were performed in liver paraffin sections of these animals. mRNA expression of PCNA (**E**), Ck19 (**F**), Sox9 (**H**) and Yap1 (**I**) was analyzed and graphed. The data were normalized for the amount of GADPH mRNA in each sample. Data are shown as mean ± SEM and graphed (****/####p<0.001;***/###p<0.001).

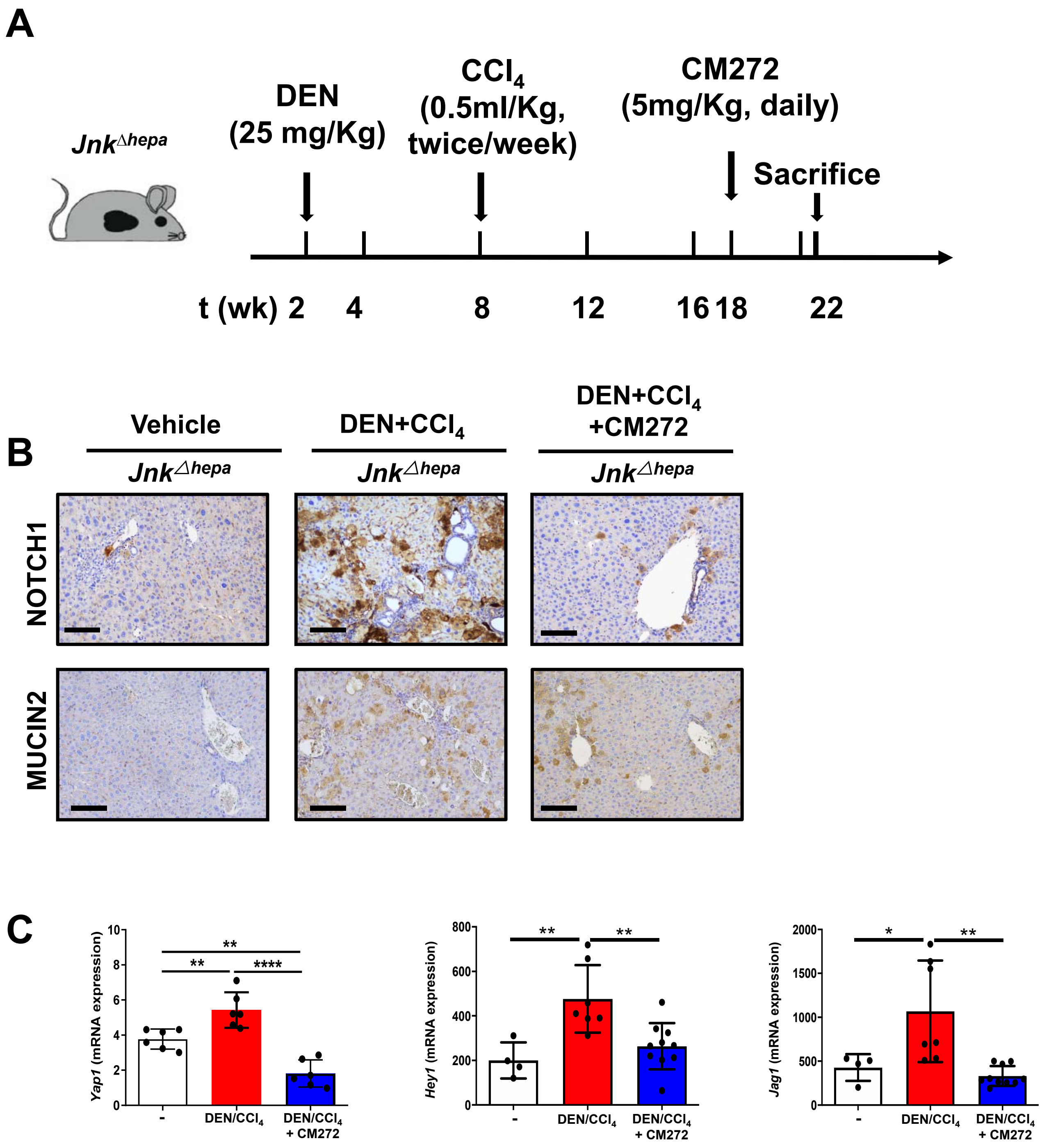

Figure S9. (**A**) Schematic representation of the animal model used. (**B**) Notch1 and Mucin-2 immunohistochemistry staining were performed in liver paraffin sections of these animals. Scale bars, 500 µm. (**C**) Expression of Yap1, Hey1 and Jag1 was analyzed and graphed. The data were normalized for the amount of GADPH mRNA in each sample. Data are shown as the mean SEM (n=5 mice per group;**p<0.01-****p<0.0001).
